# [Retracted] SOX2 knockdown inhibits the migration and invasion of basal cell carcinoma cells by targeting the SRPK1‑mediated PI3K/AKT signaling pathway

**DOI:** 10.3892/ol.2024.14635

**Published:** 2024-08-19

**Authors:** Zhuo-Ran Li, Yong Jiang, Jian-Zhong Hu, Yang Chen, Quan-Zhong Liu

Oncol Lett 17: 1617–1625, 2019; DOI: 10.3892/ol.2018.9810

Following the publication of this paper, it was drawn to the Editor's attention by a concerned reader that certain of the immunohistochemical data shown in Fig. 1A, the cell migration and invasion assay data in Figs. 2C-F and 4C-F, and immunofluorescence data in Fig. 3E and F had already appeared in previously published articles written by different authors at different research institutes, one of which has been retracted. Owing to the fact that the contentious data in the above article had already been published prior to its submission to *Oncology Letters*, the Editor has decided that this paper should be retracted from the Journal. The authors were asked for an explanation to account for these concerns, but the Editorial Office did not receive a reply. The Editor apologizes to the readership for any inconvenience caused.

